# Exploring the acceptability and experience of receiving diabetes and pregnancy care via telehealth during the COVID-19 pandemic: a qualitative study

**DOI:** 10.1186/s12884-022-05175-z

**Published:** 2022-12-13

**Authors:** S. L. Kozica-Olenski, G. Soldatos, L. Marlow, S. D. Cooray, J. A. Boyle

**Affiliations:** 1 Monash Centre for Health Research and Implementation, Monash University, Melbourne, Locked Bag 29, Clayton, Victoria 3168 Australia; 2 Diabetes and Vascular Medicine Unit, Monash Health, Melbourne, Victoria Australia; 3 Department of Obstetrics and Gynaecology, Monash Health, Melbourne, Victoria Australia; 4 Eastern Health Clinical School, Monash University, Box Hill, Melbourne, Australia

**Keywords:** Diabetes, Pregnancy care, Telehealth, Qualitative, COVID-19

## Abstract

**Background:**

The COVID-19 pandemic has significantly impacted the delivery of diabetes in pregnancy care and general maternity care. This study aimed to explore the experiences and acceptability of telehealth use in diabetes in pregnancy care during the COVID-19 pandemic, from the perspectives of pregnant women and their clinicians. The secondary aim was to explore the experiences of pregnant women receiving general maternity care via telehealth during the COVID-19 pandemic.

**Methods:**

In-depth qualitative semi-structured interviews were undertaken and analysed via thematic inductive approaches. The Nonadoption, Abandonment, and Challenges to the Scale-Up, Spread, and Sustainability of Health and Care Technologies Framework (NASSS) was applied.

**Results:**

Eigthteen interviews were conducted with culturally and linguistically diverse pregnant women and 4 clinicians (endocrinologists and dietitians). All interviewees were satisfied with telehealth as a positive alternative to face-to-face consultations for diabetes care during the COVID-19 pandemic. Numerous benefits of delivering diabetes care via telehealth were discussed and themes centred around greater access to care, economic benefits and improved safety. Most barriers concerned the adopters (clinicians), yet, feasible and realistic suggestions to overcome barriers were voiced. The scope for technology adaptation and ongoing embedment into routine diabetes care was described. Overall, a hybrid flexible delivery model, predominantly consisting of telephone consultations, with some face-to-face consultations for initial diabetes appointments was recommended for future care. The use of telehealth in replacement of face-to-face appointments for general maternity care was perceived as reducing care quality.

**Conclusion:**

In this study, telehealth was viewed as acceptable to women and clinicians for diabetes in pregnancy care, supporting the ongoing delivery of a hybrid service model of telehealth and face-to-face care. These findings provide valuable information to improve diabetes in pregnancy services to meet the needs of women during the COVID-19 pandemic and beyond.

**Supplementary Information:**

The online version contains supplementary material available at 10.1186/s12884-022-05175-z.

## Introduction

The COVID-19 pandemic has seen unprecedented changes and innovation to health services worldwide including the rapid adoption of remote consultation methods. In Australia, there has been a shift towards the growth of home-based care across all health services, as the Chief Health Officer directives have restricted attendance at acute health services [1]. This is particularly important for pregnant women who are at a higher risk of developing complications from COVID-19 [1]. Prior to the COVID-19 outbreak, the overall uptake of telehealth, including for maternity care, had been slow and fragmented [1, 2].

Maternity services needed to adapt quickly as pregnant women cannot delay childbirth and the need for hospital-based pregnancy care until the pandemic is over. In Australia, pregnancy care drastically changed during the pandemic in many areas where COVID-19 was more frequent. Many antenatal consultations have been delivered via telephone or video consultations, including for women with diabetes in pregnancy [3, 4]. Within this study, telehealth is defined as the delivery and facilitation of health and health-related services via telephone or video conferencing methods consistent with the Australian government definition [5].

Gestational Diabetes Mellitus (GDM) is the most common medical complication of pregnancy, estimated to affect around 8–30% of all pregnancies worldwide [6, 7]. The prevalence of GDM is increased in migrant women from Hispanic, South or East Asian, Pacific Islands, as well as Aboriginal or Torres Strait Islander (Indigenous) Australians compared to non-migrant populations [8]. GDM is associated with short and long-term maternal and foetal adverse outcomes [9]. Among women with a history of GDM up to 19.7% will go on to develop Type 2 Diabetes Mellitus [10]. The COVID-19 pandemic appears to have negatively impacted GDM prevalence with an increase in GDM diagnosis in 2020 compared to 2019 [11]. Moreover, the literature suggests that gestational diabetes control was lower during COVID-19 pandemic lockdowns [12], highlighting the need for future research exploring the relationship between GDM and COVID-19.

The use of telehealth in the setting of diabetes in pregnancy has been explored in two recent systematic reviews, demonstrating improvements in blood sugar control, whilst maintaining optimal maternal and foetal outcomes [13, 14]. However, few studies have been conducted to explore the views of women and their healthcare providers on telehealth for their pregnancy and diabetes care [15]. Given the significant changes to pregnancy care delivery methods used during the COVID-19 pandemic, the vulnerability of pregnant women and planned ongoing use of telehealth in many health services, exploring the acceptability and feasibility of telehealth utilisation is essential to inform sustained and improved service implementation of technology into routine practice.. This information will also support better patient -centred care, associated with improved patient experience and health outcomes [16]. The primary aim of this study is to explore the experiences and acceptability of telehealth utilisation for diabetes in pregnancy care from the perspectives of women and their healthcare professionals during the COVID-19 pandemic. This was broad across diet, exercise, weight and blood sugar measurements and with a variety of clinicians. The secondary aim was to explore the experiences of pregnant women receiving general maternity care during the COVID-19 pandemic.

The findings of this study will provide critical insights to guide and reorientate models of care, exploring whether telehealth could be a useful adjunct to routine care for women with diabetes in pregnancy beyond the COVID-19 pandemic.

## Participants, ethics and methods

### Study design and theoretical approach

This article reports on the qualitative descriptive component of a mixed-methods study examining the experiences of English speaking pregnant women and health professionals during the COVID-19 pandemic. The larger mixed-methods study included a quantitative survey with pregnant women able to speak English (*n* = 100) and qualitative interviews with overseas born non-English speaking pregnant women and hospital-employed interpreters. Given the volume and depth of data collected, the qualitative interviews with non-English speaking pregnant women and interpreters, as well the quantitative survey data is to be published elsewhere.

**Fig. 1:**
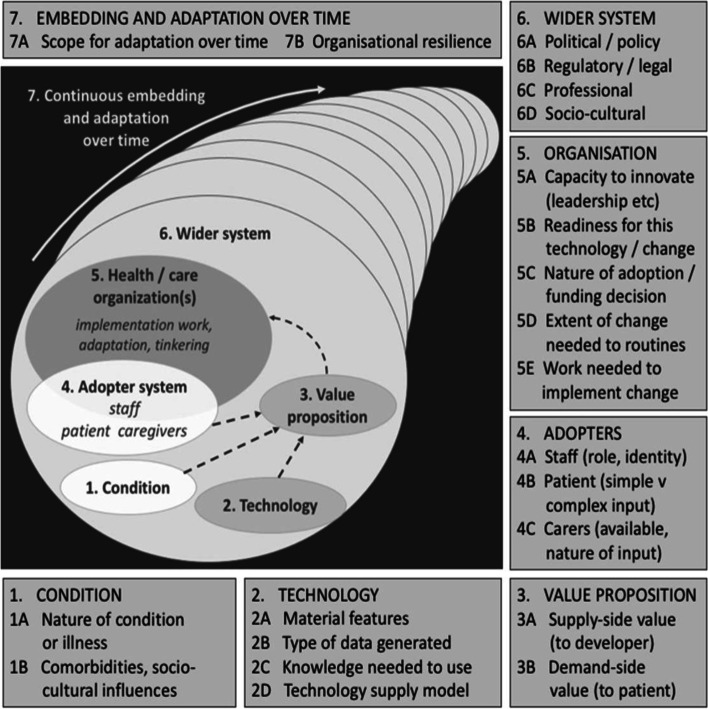
the Nonadoption, Abandonment, and Challenges to the Scale-Up, Spread, and Sustainability of Health and Care Technologies Framework (NASSS) written by Greenhalgh et al. Figure used with permission from©Trisha Greenhalgh, Joseph Wherton, Chrysanthi Papoutsi, Jennifer Lynch, Gemma Hughes, Christine A’Court, Susan Hinder, Nick Fahy, Rob Procter, Sara Shaw. Originally published in the Journal of Medical Internet Research (http://www.jmir.org), 01.11.2017 (Reference [18]). This is an open-access article distributed under the terms of the Creative Commons Attribution License (https://creativecommons.org/licenses/by/4.0/), which permits unrestricted use, distribution, and reproduction in any medium, provided the original work, first published in the Journal of Medical Internet Research, is properly cited. The complete bibliographic information, a link to the original publication on http://www.jmir.org/, as well as this copyright and license information must be included.

This evaluation was guided by the Nonadoption, Abandonment, and Challenges to the Scale-Up, Spread, and Sustainability of Health and Care Technologies (NASSS framework) [17, 18]. The NASSS framework is a multi-level theoretical framework designed to assess acceptability, uptake and challenges of technology innovations within health care settings. It considers seven domains: 1) the condition, 2) the technology, 3) the value proposition, 4) the adopters, 5) the organisation, 6) the wider system, and 7) embedding and adaptation over time (Fig. 1). As the study intended to inform improvements in clinical care, the theoretical underpinning of the study is one of pragmatism [19].

### Study setting

This study was conducted at the Monash Health Maternity Service across the Clayton, Dandenong and Berwick Campuses, between May and September 2021. Monash Health is one of Australia’s largest maternity services and is located in a major area of refugee resettlement, situated in the South-eastern suburbs of Melbourne Victoria. During the COVID-19 pandemic, Australia’s federal, state and territory governments introduced lockdown restrictions that commenced in March 2020 and continued into 2021 in order to slow the spread of COVID-19. This included a 262 day lockdown which included restricted attendance at acute health services, cancellation or postponement of elective surgeries, limited access for hospital visitors and the closure of schools and businesses (https://www.australia.gov.au). Subsequently, during this time period diabetes care was delivered predominantly via telephone consultations with face-to-face delivery commonly utilised for the initial diabetes assessment and education session, as well as if insulin education was required. Video conferencing was utilised in some cases for insulin education sessions. Women’s blood sugar level (BSL) readings were emailed to the diabetes administration teams by the women; automated transmission of glycaemic nor weight data was not available. Women received their maternity care via a combination of telephone, videoconferencing and face-to-face consultations. Pregnant women deemed as having a ‘high-risk pregnancy’ attended the Maternity service in person. The health service plans to continue using telehealth for pregnancy and diabetes care. This study was approved by the Monash Health Human Research Ethics Committee. Participants were provided information about the study and informed verbal consent was obtained from all subjects prior to participation, as approved by Monash Health Human Research Ethics Committee.

### Population group and sampling

#### Pregnant women with diabetes

All pregnant women diagnosed with diabetes (type 1 diabetes mellitus [T1D], type 2 diabetes mellitus [T2D] and GDM) who attended the Monash Health Maternity Service were invited to complete an anonymous online survey from April to July 2021. At the end of the online survey, women were able to express their interest in participating in qualitative interviews. These women were contacted by the research team and provided written study information. Women were required to speak English. Verbal consent was obtained and participation was voluntary. A maximum variation sampling approach was utilised to capture the perspectives and experiences of a broad range of women from diverse backgrounds. Eigthteen women were interviewed; 35 women expressed interest in the study via the online survey, 12 women were unable to be contacted, 4 women had delivered their babies and 2 women declined participation due to complex mental and social issues including housing insecurities and severe anxiety.

#### Clinicians

Endocrinology doctors (specialists, residents and registrars), diabetes educators and allied health professions employed within the Diabetes in Pregnancy Service at Monash Health, at the time of COVID-19 were invited to participate in the study. Potential participants were identified with assistance from senior management and recruited through a direct, individualised email from the researcher team. The voluntary nature of these interviews was explicitly stated and verbal consent was obtained.

### Data collection

Interviews were conducted via telephone by two researchers between May to July 2021, guided by an interview schedule developed by the authors who are across various relevant clinical disciplines (dietetics/ endocrinology/ diabetes education/ obstetrics) and informed by a review of previous literature. The interview guides were pilot tested prior to use with a consumer group of recently pregnant women (*n* = 6) and clinicians (*n* = 3), and questions and wording modified in accordance with their feedback. Questions elicited information about women’s and clinicians’ experiences using telehealth, advantages and disadvantages of telehealth, and suggestions to improve diabetes in pregnancy and maternity service in the future. See Additional file 1: Supplementary file 1.

All interviews were audio-taped using a digital recorder, de-identified upon conclusion and transcribed verbatim by an independent transcribing service or via computer real-time transcription software, Otter.ai Inc. (Mountain View, CA). All transcripts were checked for accuracy by researchers. Data saturation was confirmed for interviews with pregnant women and determined when no new ideas emerged from the interviews. There was a limited number of clinicians interviewed due to the stress of COVID-19 on the workforce,. However, the interviews generated a rich dataset with complex insights into healthcare delivery by telehealth and saturation and consistency of themes was met Descriptive participant characteristics were collected via the completion of a brief demographic survey at the commencement of interviews.

### Data analysis

De-identified transcripts were analysed independently by two experienced female researchers using thematic and inductive analysis approaches, allowing coding and categorising of primary patterns within the data. Initial codes were assigned and similar topics were grouped into categories and reviewed to produce themes. Qualitative researchers met regularly to discuss emerging patterns, overarching themes and create a visual representation. Themes were organised in accordance with the NASSS frameworks. In-depth discussion of themes took place amongst investigator team members before a final iteration of results. Thematic coding of data and development of models were assisted by Dedoose Software (web application for managing, analysing, and presenting qualitative and mixed method research data (2018). Los Angeles, CA: Socio Cultural Research Consultants). Verbatim quotes which represented research findings for each theme were highlighted. This study was conducted and reported according to the Consolidated criteria for reporting qualitative studies (COREQ) guidelines [20].

## Results

The results from the qualitative interviews with women and their clinicians are presented under each of the seven domains of the NASSS framework. Table 1 applies the NASSS framework to the diabetes in pregnancy telehealth service.

**Table 1:** The NASSS framework assessment of the diabetes in pregnancy telehealth service

**1**	**Condition:**	
1A	Diabetes in pregnancy	simple
1B	Co-morbidities: age, multiple co-morbidities; Sociocultural: English proficiency, health literacy,	complicated
**2**	**Technology:**	
2A	Material properties: Telehealth or phone. Variable issues with audio/video	simple/complicated
2B	Knowledge generated by it: health information and BSL provided	simple
2C	Knowledge to use it: telephone use viewed as simple but technology issues reported for video conferencing	complicated for video conferencing
2D	Supply model: Health service provided or personal mobile phone	simple
**3**	**Value proposition**	
3A	Supply side: Decreased COVID exposure/efficiency/ maintenance of service	simple
3B	Demand side value: Decreased COVID exposure, convenience, access to care in setting of pandemic, reduced cost	simple
**4**	**Adopters**	
4A	Staff: Clerical and medical staff need to learn new skills. Increased paperwork during consultations	complicated-
4B	Patient: variable-dependent on care need, health and technology literacy. Less satisfied for maternity care to be received via telehealth	complicated for maternity video/ simple for telephone use
4C	Carers: assumes a caregiver will be available when needed	complicated
**5**	**Organisations**	
5A	Capacity to innovate in general: accelerated by COVID	simple
5B	Readiness for this technology: enhanced readiness in the setting of COVID directed need	simple
5C	Nature of adoption and/or funding: health service directive to transition to telehealth in response to COVID. Change to government funding regulations to enable reimbursement	complicated
5D	Extent of change needed to organisational routines: Change from in person consultations to mostly telehealth. Development and dissemination of telehealth guidelines for staff	complicated
5E	Work needed to plan, implement and monitor change: Clerical and medical staff worked to triage appointments types and communicate with patients.	complicated
**6**	**Wider system**	
6A	Political/ policy context: Government directives regarding change to funding of telehealth, provision of PPE as required	simple
	Professional bodies: Guidelines developed regarding management. Professional bodies supportive	complicated
	Sociocultural context: issues regarding equity of access for non-English speaking women/ use of telehealth	complicated
**7**	**Embedding and adaption over time**	
7A	Scope for adaption over time: proposed hybrid model of in-person and on-going telehealth consultations. Need for ongoing funding model of telehealth.	complicated
7B	Organisational resilience: Organisation continues to adapt to uncertainties related to COVID	complicated

Table 2 describes pregnant women’s demographic characteristics. Eigthteen interviews were conducted with pregnant women with the majority being diagnosed with GDM and many having had prior GDM (prior GDM =8; No prior GDM = 8 and T1D/T2D *n* = 2). The mean duration of the telephone interviews was 33 minutes (range: 21 to 50 minutes). Women were from culturally and linguistically diverse backgrounds. More than half of the women were born outside of Australia and some of the countries of birth included India, Pakistan, Afghanistan, France, China and Vietnam. All these women spoke English.

**Table 2:** Demographic characteristics of pregnant women with diabetes

Characteristics	Participants (*n* = 18)
**Age: mean (SD)**	34.35 ± 4.57
**Pregnancy gestation**	
< 27 weeks	3
28-34 weeks	8
35+ weeks	7
**Number of children at home**	
0	4
1	6
2+	8
**Prior GDM**	
Yes	8
No	8
Type 1 Diabetes Mellitus /Type 2 Diabetes Mellitus	2
**Australian born**	
Yes	8
No	10
**Years living in Australia**	
Australian born	8
< 2 years	1
2-5 years	2
6-9 years	3
10 years or more	4
**Language spoken at home**	
English	8
Language other than English	10
**Household**	
Living with spouse	18
Living without spouse	0

Four clinicians were interviewed (3 endocrinologists and 1 dietitian). The mean duration of the telephone interviews was 33 minutes (range: 26 to 36 minutes). The endocrinologists had all worked within their current role for 1-5 years and within their profession for more than 10 years. The dietitian had worked in their current role for 5 years and within the profession for more than 10 years. All had delivered diabetes care via telehealth during the COVID-19 pandemic. Illustrative quotes are reported in Table 3.

**Table 3:** Summary of NASSS framework domains and key quotes

NASSS domain	Themes	Quote
The condition	Disappointed with diabetes in pregnancy diagnosis	“So when I got diagnosed with it this second time, I wasn’t surprised, but I think you’re always disappointed you know, you’re always a bit, not devastated, but obviously wish you didn’t have it” (Participant #3) “I was really disappointed that I have it. Because I knew from my friend, that it’s a little bit difficult… I am also scared of needles” (Participant #6)
The technology	Technology challenges using videoconferencing	*“So I ended up how to just under normal phone call. So yeah, I think video chatting would be good but their internet connection wasn’t good. A little bit annoyed me. It was kind of like moving the car and something because you don’t know”. (Participant #8)* *“It took an hour and a half trying to figure out how to get on to the meeting. It would have been easier if they would have just called me” (participants #1)*
Value proposition	*Greater access to care and convenience* *Safety* *Share clinician load across various health care sites* *Time efficiency and economic benefits* *Meaningful employment*	*“Telehealth is helping if they don’t have to do any measurements or anything, like it saves you from travelling, they give you the same information. If you’re sitting in the room, you don’t have to, like the waiting room in that hospital” (Participants #5)* *“Because of this COVID pandemic, there are so many points [advantages], like what should I say during pregnancy your immunity it low. So it’s better to have a telehealth appointment, because we don’t need to go there and exposing other patients and getting that infection” (Participant #2).* *“The other best benefit of it, I think is, if someone is running behind, we could actually help them offsite. For example, if I work in the Dandenong Clinic and they tell me that Monash Link and Clayton, they are running behind. I can actually say, “That’s okay. I’ll call the patient from Dandenong”(Endocrinologist).* *Regarding the general flow, I do find that phone consultations are a lot smoother. The reason being, when patients come in face to face, they usually bring in their other children, sometimes they’ve got their family members or support person there with them, which is lovely, but the waiting room becomes extremely crowded. And waiting for patients to go from one end of the clinic, of the waiting area, to go into your clinic room, that sometimes takes a while.” (Endocrinologist).* *“It feels good to be getting to do something important and providing care to people that need it even despite what’s going on in the world. I guess you feel like you’re doing something that’s still very meaningful and I think when restrictions were tight and so I think patients were clearly grateful that we were providing the service that we needed” (Endocrinologist).*
Adopters: women with diabetes	*Positive telehealth experience*	*“It has been really great. I’ve only had telehealth appointments for my gestational diabetes. And they’ve been totally fine. Diabetes, I think it’s the thing we can check and discuss over the phone. So it’s not that difficult. But Maternity care should be like, it is better to have face-to-face. (Participant #7)* *“They just said you’ll need to get your blood pressure done and I was like how the hell am I going to do that? And they were like just go to your GP. And that’s a bit silly, because I’m still gonna go to a doctor anyway. My GP measured my tummy and she did the foetal heartbeat and my blood pressure” (Participant #3).*
Adopter: clinicians	*Initial phase of pandemic was challenging* *Increased administration workload* *Perceived reduced value of telehealth* *Telehealth is not ideal model for all pregnant women*	*“The initial phase was quite a time of uncertainty. Uncertainty with the processes, with the procedures, with the co-ordination and communication between the teams, the diabetes educators, the endocrinologist, the administration.” (Dietitian).* *“The most time-consuming part for the doctors at this point is sending out a script, or sending out pathology forms to the patients. We have to print out the patient’s script, phone up the patient’s usual chemist, which some patients know, some patients don’t know, and they would Google it…Then we write it down, and our admin staff will have to email that script to the chemist. So that is very time consuming.” (Endocrinologist)* *“Clients are not valuing the consultation as much as they previously had…. So maybe seeing it as not as important or seeing as the optics are different, you know you’re with someone you don’t know, you haven’t seen, particularly with a telephone consult you don’t see face to face. Some person you’ve never heard of is just calling you” (Endocrinologist).* *“There are patients who are illiterate and can’t document their sugar levels down and we tend to just go through the Glucometer ourselves. And during over the phone, they really can’t tell us what their sugar levels are. They also heavily rely on families or friends to help them document and email through and I think that’s extra stress for them. I don’t know what is the best way to help this to, but illiterate... Illiteracy patients I think will need some extra support” (Endocrinologist).*
Organisation	*Professional isolation*	*“You know if I really needed help or wanted to a second opinion I could always seek it. But I think there’s less of the kind of corridor conversations that were really good with colleagues both in terms of advancing clinical knowledge, working out management plans for patients, but also just making sure that your colleagues are okay. Just small talk and how are you and that sort of thing. So, I think that has fallen away quite a lot. And I think that’s a real shame… I do at times feel a bit lonely in clinics” (Endocrinologist).* *“Many of my colleagues are working from home. So I think there’s a less of a team – you lose that sense of a team when you’re normally in a clinic there’s a few clinicians, there’s that sense of working as part of a team and there are clinicians available that you can discuss tricky cases with very easily. It lends itself to that because if someone’s physically there. You can knock on the door and have a chat about it” (Endocrinologist).*
Wider system	*Cultural factors*	*“I think because you don’t have the confidence to listen to the English on the phone. More hard. There is no body language involved. Yet so yeah, I think they might prefer to face to face or they can even bring something to play with.” (Patient #12)* *“For less health literate patients…I think visual or cues from body language and so on are more important and I think for that situation face to face appointment is necessary” (Endocrinologist).*
Embedding	*Future scale-up: delivery of a hybrid diabetes in pregnancy model with initial session and insulin education delivered in person*	*“If I am a patient, my ideal scenario would be your first appointment will be face-to-face, so you can ask a little bit more questions. And some patients are very anxious when they’ve got gestational diabetes (Endocrinologist*). *“Like if someone like myself has never had a sugar check-up and that machine and feels the needle and all that ….it will be I think better for the face to face. And they can talk to someone and have the confidence back. I got stuck on the first one, like the blood wasn’t coming. Maybe I was scared that I wasn’t pointing to the right spot. But then she came and showed me how to do it.” (Participant #10)* *“Face-to-face delivery if starting insulin. That means that she can come in, see us and then see the diabetic nurses, all in the one hit, That would work for her, save her a trip to pharmacies, save the calling her and have to do a separate consultation. And I think with COVID pregnancy which is pretty terrifying for some women. It’s a lot better done face to face (Endocrinologist).*

### The condition

Overall, 16 women interviewed were diagnosed with GDM, 1 with TDM1 and 1 with TDM2. Women reported initial disappointment and anxiety when first diagnosed with GDM.

“I was really disappointed that I have it [GDM]. Because I knew from my friend, that it’s a little bit difficult… I am also scared of needles” (Participant #6).

Women reported variability in their GDM diagnosis notification with some told via telephone by their GP and others notified in person via midwives at the hospital.

### The technology

Technical functionality and reliability of telephone and video conferencing.

Almost all women interviewed reported confidence in their use of technology. Few reported using telehealth (telephone or video) for medical care before the COVID-19 pandemic. Women indicated that clear instructions for telehealth appointments had been provided for both maternity and diabetes care. Variable responses were reported by both women and clinicians regarding the quality of the video conferencing technology. Difficulties with internet quality and connection, as well as audio and video clarity resulted in interrupted care. However, others interviewed reported high satisfaction with video conferencing technology.

Telephone consultations resulted in fewer technology barriers for women, however, barriers to accessing private consultation rooms, headphones and hands-free telephones were discussed by clinicians.

### Value proposition

Within the context of the COVID-19, a highly contagious and potentially fatal infection, the risk-benefit balance of employing telehealth was clear and all interviewees supported the use to various extents.

All women described benefits of utilising telehealth and these benefits could be grouped into (a) greater access to care, (b) reduced personal costs and (c) improved safety. Greater access to care focused on telehealth benefits such as convenience, whereby, women did not have to leave their homes and this was particularly valuable for women with other children. Women also highlighted that telehealth allowed greater access to diabetes in pregnancy care specialists for those living in rural areas. Reduced personal costs included reduced disruptions to women’s work/professional lives (reduced wage losses), travel, as well as parking expenses. Improved safety related to reduced COVID-19 exposure risk, as many viewed attending hospital outpatients’ clinics as a “high risk” setting.

Some clinicians reported that telehealth utilisation reduced the number of women who failed to attend appointments and improved their ability to engage with “harder to reach” women who “often fall through the cracks”. Their rationale was that telehealth is more convenient and minimises barriers to attending appointments such as effort, costs and time.

Telephone consultations were also viewed as time-efficient and increased the numbers of women that clinicians could review in clinics. This was thought to be due to the minimisation of interruptions and time associated with manoeuvring through crowded waiting rooms. Another advantage of telehealth highlighted by clinicians was that it provides potential opportunities for clinicians to share patient loads across multiple hospital sites, particularly if one site is overwhelmed by high volumes of patients. Clinicians also discussed that the rapid changes achieved during the pandemic illuminate the future possibilities for further adaption and refinement of delivery methods.

“It’s opened up a whole aspect of not doing things the way that we’ve always done them and perhaps we can look at some different models of? …So I think that that’s probably a plus side of things of enabling us to look at how we can do these things better” (Dietitian).

### Adopters

#### Women’s experience using telehealth for diabetes in pregnancy care

Overwhelmingly, all women interviewed described a positive experience utilising telephone consultation for their diabetes care. Frequently women reported initial concerns receiving diabetes care via telephone, however, their anxiety reduced once care was commenced. Delivery of the initial diabetes education session in person was viewed as favourable, particularly amongst women without prior GDM diagnosis, as women felt more comfortable asking questions and wanted feedback on their technique for testing their blood sugar levels. All interviewees described confidence in their ability to manage their diabetes through telehealth support provided by the diabetes team.

Few barriers were described by women in relation to receiving their diabetes care via telehealth and many women interviewed (over half) had no suggestions for diabetes service improvement. However, barriers described by women included challenges receiving prescriptions for insulin and this was viewed as particularly challenging for women whose first language was not English. Approximately one-third of the women discussed issues contacting the diabetes educators outside of hours, lengthy appointment wait-times and inadequate access to nutrition and lifestyle advice.

#### Carers experience

Almost all women reported that the use of telehealth appointments did not impact their partner’s involvement or attendance. Women revealed that their partners were often unable to attend appointments regardless of the mode of delivery due to work commitments.

#### Clinicians experience

There was agreement that the initial phases of the pandemic were challenging for clinicians, due to the uncertainty of the virus and their limited prior experience utilising telehealth. Despite this, clinicians reported being “agile” and able to rapidly change their methods of delivering care to promote a “smooth transition”. All clinicians interviewed perceived telehealth to be a valuable initiative that enhanced access to diabetes care. However, challenges to adapting to telehealth service delivery were reported.

##### Clinician challenge: increased administrative workload associated with pharmacy and pathology requests

Challenges such as increased administrative work load were described, particularly concerning scheduling and coordinating women’s reviews, as well as writing paper based prescriptions for insulin and pathology forms. Paper based processes were viewed as sub-optimal and “burdensome” with delays frequently reported. Delays of prescriptions were particularly concerning for women requiring insulin, as delays in commencement of treatment were viewed as a clinical risk.

Other challenges included clinicians unable to view women’s blood sugar level booklets or glucometers in person. This challenge was compounded as many women were emailing the clinic their BSL readings, some did not and others went missing. This increased the length of appointments as women needed to read out their recent BSLs, causing frustration for both patients and clinicians. This was increasingly an issue for women that had low literacy or did not speak English. Clinicians recognised the increased workload and challenges undertaken by the administration team to follow-up blood sugar levels, pathology results and prescriptions. Many felt that the administration team had been very supportive and “they did the heavy lifting that made this (telehealth delivery) possible”.

##### Clinician challenge: issues contacting and communicating with women

Clinicians described that women were often distracted during the telephone consultations and were multitasking. Issues with women forgetting about their appointment were also commonly described. Phone calls from the hospital and staff are displayed as private caller (no phone number) on the recipient’s phone. These are often associated with spam calls and therefore some women did not answer these calls whilst others had poor phone reception.

##### Clinician challenge: women’s perceived value of telehealth

Clinicians suggested that some women appeared to place reduced value and accountability on telephone consultations, in contrast to attending face-to-face appointments. Furthermore, challenges keeping to scheduled appointment time due to high patient numbers were also described. Clinicians felt that women were less understanding about appointment delays, as women could not physically see the crowded waiting rooms and demand for diabetes care.

#### Women’s experience of using telehealth for maternity care

Most women interviewed highlighted a strong preference for maternity care to be delivered face-to-face and perceived telehealth as compromising their care. Women described that face-to-face care delivery improved their confidence and valued the physical reassurance provide by clinicians during physical check-up including blood pressure being taken, fundal height measured, and growth of the baby checked (ultrasounds).

“I feel much better if I am going to the hospital. Much better” (Participant #2).

Over half of the women interviewed reported not self-monitoring their weight, blood pressure or fundal height because they did not have appropriate equipment or did not feel comfortable or confident. Additionally, one-third of women reported going to GP to have these measurements taken as a result of not being able to attend maternity appointments in person.

Some women interviewed highlighted that they were satisfied with having their early pregnancy appointments via telehealth and this was especially amongst women who had other children. These women highlighted the convenience, comfort and safety of having appointments from home, particularly if they were struggling with illness during the early stages of their pregnancies. However, they highlighted the need for a combination of both telehealth and face-to-face appointments.

**Fig. 2:**
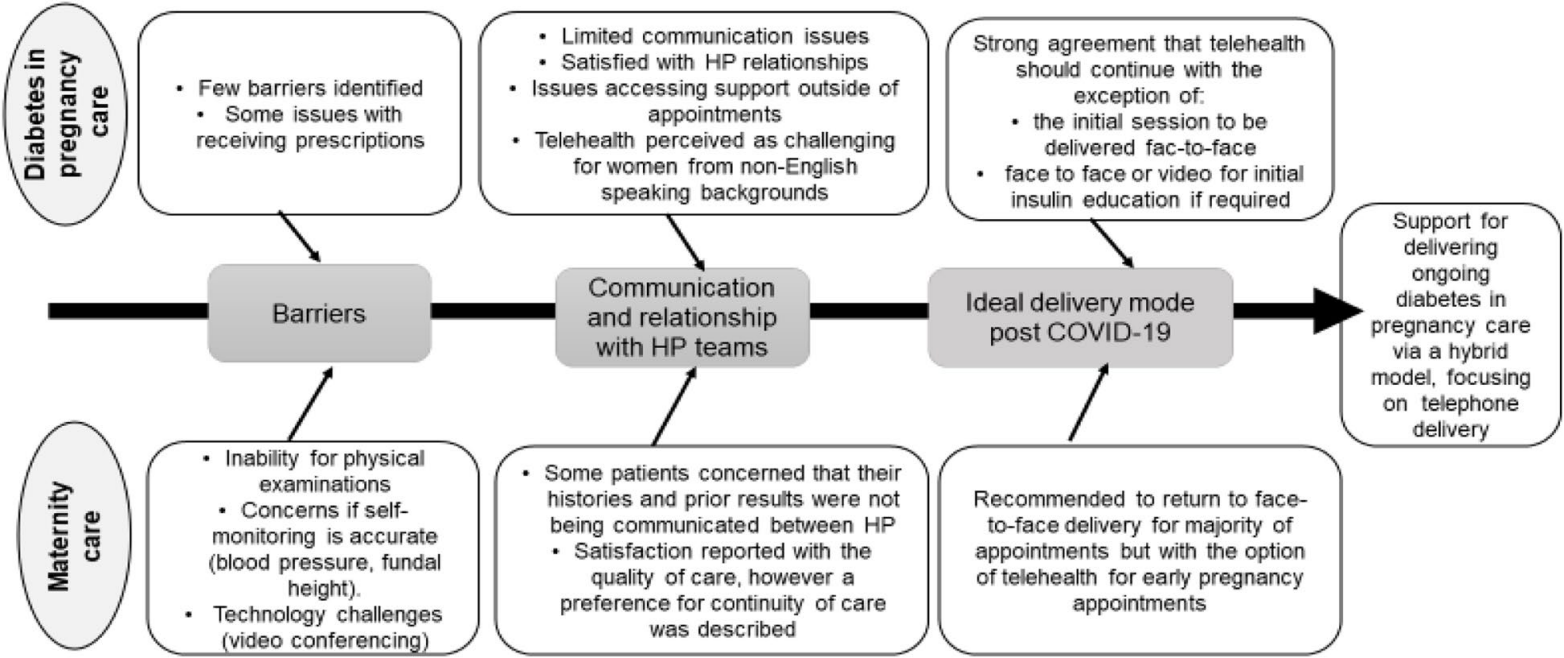
The values of telehealth utilisation in the setting of maternity care and diabetes care from pregnant women’s perspectives

A minority of women felt rushed and that the telehealth appointments were shorter as a result of “lost personal connections”. Another key barrier described by women was lack of continuity of care in relation to both their midwives and obstetricians. However, women described that they were satisfied with the maternity care provided given the unprecedented circumstance. A comparison between telehealth experience and acceptability for diabetes and maternity care is described in fig. 2 from the women’s perspectives.

### Organisation

All clinicians recognised and appreciated the organisational challenges associated with the rapid implementation of telehealth models of care.

I think the situations constantly changing. I think overall our organisation has done really well” (Endocrinologist).

A diversity of perspectives was reported by clinicians regarding their satisfaction with the organisational support received. Some felt well supported and given the urgency of the situation believed appropriate training and supports were available, as well as flexible working arrangements (working from home for some). However, most of the medical team continued to work onsite.

In contrast, others described that organisational supports could have been improved in relation to infrastructure and telehealth resources. Barriers accessing resources required for telehealth such as headphones, functioning microphones, lighting, screens and working sound systems were voiced for videoconferencing. One clinician reporting that a project manager would have been ideal to support initial set-up and for troubleshooting. Identified lack of hospital wide protocols surrounding procedures for gaining formal consent from women, ID checks and privacy requirements as gaps in the initial setups. Others reported that organisational efforts could have been improved to boost staff moral and address staff feeling socially isolated.

#### Issues communicating with the team and professional isolation

As a result of the need for social distancing during the COVID-19 outbreak, communication within the diabetes team was described as more challenging. Clinicians discussed that the sense of a team and access to support from colleagues was also disrupted with some staff feeling less connected to their colleagues. Professional isolation was discussed by most interviewees. Reduced team meetings, corridor conversations and access to senior clinicians on site also led to fewer learning opportunities, particularly for more junior staff.

“Many of my colleagues are working from home. So I think there’s a less of a team – you lose that sense of a team when you’re normally in a clinic there’s a few clinicians, there’s that sense of working as part of a team and there are clinicians available that you can discuss tricky cases with very easily. It lends itself to that because if someone’s physically there. You can knock on the door and have a chat about it” (Endocrinologist).

### Wider system

The rapid implementation of telehealth models of care was consistent with the wider organisational guiding principles of consistently providing safe, high quality and timely care to all.

#### Cultural factors

Health professional agreed that the challenges of working with women who did not speak English or who had very low health literacy was amplified when utilising telehealth. Clinicians described working with these women as often time consuming and logistically challenging. For women that spoke common languages where hospital employed interpreters were available this process was easier, however, for women that spoke a language that required a third-party service significant time delays were described.

“Labour-intensive….to organise an interpreter for a consultation time that suits that patient and us…it is very clunky [challenging]” (Dietitian).

### Embedding and adaption over time

#### Future care (ideal model of care delivery) for the diabetes service

All clinicians and pregnant women with diabetes recommended the continuation of telehealth within the diabetes pregnancy service.

“I think we’ve learnt that (phone consultations) are quite optimal and probably might be the best way to do it in future. Even when we’re not limited by COVID restrictions” (Endocrinologist).

Offering a hybrid model into the future was unanimously agreed upon by all as the ideal delivery model. The ideal model for diabetes care would include the initial appointment and education session to be delivered in person with subsequent reviews via telephone consultations with the exception of commencement of insulin. Women wanted the initial appointment to be delivered face-to-face as they commonly felt nervous about their diabetes diagnosis and wanted to learn new skills of blood sugar testing in person and collect required testing equipment. Clinicians also highlighted the need for the initial appointment to be delivered in person, to ensure that appropriate diabetes education had been provided and understood by women.

Continuing to deliver subsequent consultations via telephone rather than video was preferred by clinicians, given high patient volumes and increased time associated with videoconferencing.

“Certainly, for just pure gestational diabetes telephone is easier because the consults are quite brief and our clinics are really heavily booked…logistically logging on to see them all on video would be difficult just because there’s often technical difficulties…And you also just don’t need it (Endocrinologist).

Women also consistently preferred telephone consultations for review appointments. Due to greater convenience, reduced wait times and increased flexibility as women did not need to stay home and wait in front of their computers. Additionally, some women reported being shy and preferred that clinicians did not see inside their houses.

Face-to face delivery for the education of insulin commencement was recommended by women and clinicians. However, many women suggested video conferencing would be adequate if face-to-face delivery was not possible.

However, women and clinicians clearly stated that choice should be given to women to access face-to-face appointments if requested, if women had low health literacy, interpreter requirements or were perceived as high-risk. Acknowledging women’s preferences and choices to inform delivery modes was repeatedly viewed as important by both women and their health providers.

“I do think that patient’s preferences needs to be factored in as well” (Endocrinologist).

#### Suggested improvements for future diabetes services

##### Greater administration support and access to telehealth infrastructure

Clinicians suggested improvements included greater administration support, adequate infrastructure and resources (headphones, computers with video conferencing ability and private spaces) to conduct telehealth consultations. Women discussed the need for improved appointment time management.

##### Improved workflow processes (prescription and pathology writing processes)

All clinicians highlighted the need to refine the workflow processes of writing patients’ prescriptions and pathology requests when utilising telehealth. Utilising electronic prescription methods whereby prescriptions are sent by text or email was viewed as ideal and time efficient. The use of electronic prescriptions was also supported by women to improve timely access to prescriptions.

##### Improved access to allied health and diabetes educators outside of appointment times

Greater nutrition access and input was suggested by women. Improved access to diabetes educators outside of appointment times was also recommended, as some women reported concerns with their BSL outside of their appointment time and issues accessing appropriate support.

#### Future care (ideal model of care delivery) for the maternity service

Overall, most women wanted maternity care to return to face-to-face delivery, following COVID-19 restrictions being released. Notably, a smaller number of women, mostly those with children already, said they wanted the option to choose between face-to-face or telehealth for early pregnancy appointments.

Other maternity service improvements discussed from the perspectives of pregnant women included improved organisation of clinics, reduced appointment wait-times and better-hand-over between health care providers to avoid repeating information multiple times. Women further highlighted the benefits of continuity of care to minimise inconsistent advice from various health providers and concerns about information not being passed on between clinicians.

## Discussion

This qualitative study provides insights into the experiences and acceptability of telehealth use for diabetes during pregnancy and maternity care during the COVID-19 pandemic. All clinicians and pregnant women with diabetes interviewed supported the continuation of telehealth within the diabetes pregnancy service. Numerous benefits of delivering diabetes care via telehealth were discussed and themes centred around greater access to care, economic benefits and improved safety. Most barriers concerned the adopters (clinicians), yet, feasible and realistic suggestions to overcome barriers were voiced. The scope for technology adaptation and ongoing embedment into routine diabetes care was described. A hybrid delivery model predominately consisting of telephone consultations with some face-to-face consultations was recommended for the future. The need for flexible service delivery models with women’s preferences accounted for (women centred care) was emphasised as part of a wider scale-up. The use of telehealth in replacement of face-to-face appointments for general maternity care was overall perceived as reducing the quality of care. However, women with children may prefer telehealth in early pregnancy.

Whilst the utilisation of telehealth for diabetes in pregnancy has been shown to maintain safe maternal and fetal outcomes [13, 14], to our knowledge, this is the first study to explore the impact of COVID-19 and changes to models of care within a diabetes and pregnancy service from the perspectives of women and clinicians. Consistent with findings from a scoping review exploring models of incorporating telehealth into obstetric care during COVID-19, telehealth benefits included minimising COVID-19 exposure, convenience (travel), reduced attendance burden (wage losses and childcare assistance) and continuity to provide high-quality care during the pandemic [21]. Barriers reported here were also consistent with prior literature and included limited availability of equipment and infrastructure challenged care delivery in this study [21]. Utilising electronic prescription technology was viewed as key to improving the efficiency of telehealth delivery. Clinicians interviewed viewed challenges experienced as relatively simple to address and an abundance of research has been released to optimise telehealth utilisation [22].

Guiding principles to optimise telehealth success emphasises the importance of clinicians allocating appropriate preparation time, ensuring familiarity with technologies, equipment (headphone, screen sharing if needed), professionalism (finding a suitable and quiet space) and patient-centred (decide on appropriate delivery methods such as audio only or with video conferencing, and sending information/instructions in advance) [23]. Utilising these principles would likely improve telehealth embedded within the study setting in the future.

Our findings are consistent with other Australian and international maternity care studies conducted during the COVID-19 pandemic, which found that pregnant women perceived the impacts of COVID-19 and telehealth as compromising their maternity care ( [4, 24, 25]. Findings from both national and international cross-sectional surveys of women found that pregnant women felt distressed, unsupported, alone and were dissatisfied with the evolving changes in maternity care models during the pandemic [4, 26, 27]. Limited face-to-face contact from clinicians left many pregnant women feeling “robbed” of their anticipated and desired maternity care experience [4] and elevated stress levels were reported [27, 28]. In the height of the pandemic, many women reconsidered their maternity care and there was a move towards community-based options for maternity care and a trend towards women accessing private care including from a private obstetrician within a private hospital or from midwives in private practices [28].

### Study implications

Moving forward, health services are turning their attention to how diabetes care should be delivered post-pandemic with telehealth consultations certain to be central to reorientation [29, 30]. Findings from this study suggest that there are some consultations that will require face-to-face support; such as the initial consultation and insulin commencement if required. Additionally, while telehealth use was supported for diabetes reviews by women from culturally and linguistic backgrounds in this study, the utility and acceptability amongst women from non-English speaking backgrounds was raised as a concern and remains a research gap. Clinicians highlighted the telehealth challenges of communicating with women with low health literacy and when utilising interpreters. Consistent with prior literature women with low health literacy are also more likely to struggle with maternity care system navigation [1, 2], potentially be less proactive with their maternity care and these challenges are likely to be exacerbated when utilising telehealth e.g. remembering instructions, lost insulin scripts. These findings support the need for clinicians to advocate for the tailoring of health care delivery models to individual woman’s needs and preferences, ensuring women centred care is delivered.

Moreover, the acceptability and satisfaction of telehealth use for diabetes in pregnancy and maternity care from the perspectives of overseas women that do not speak English remains largely unknown [19]. Addressing this research gap, we have undertaken qualitative interviews with women from non-English speaking backgrounds with the assistance of interpreters and have interviewed hospital-employed interpreters. These findings will be presented in future manuscripts, as well as quantitative survey data exploring telehealth acceptability from a larger sample size.

### Strengths and limitations

Whilst qualitative research does not aim to be representative it is important that research contributes to health equity and there are clear limitations to telehealth for women who do not speak English who are not included in this manuscript. Moreover, due to the stress of COVID-19 on the workforce, engagement of clinicians was extremely challenging and limited the availability of respondents. The strengths of this study are that it explores the utilisation of telehealth from both pregnant women’s and clinicians’ viewpoints. Further, the women who participated are from a wide range of culturally and linguistically diverse backgrounds reflective of the women accessing the maternity service.

## Conclusion

COVID-19 has catalysed alternative models of delivering healthcare globally. This study highlights that telehealth consultations are acceptable and satisfactory to women and some clinicians for the delivery of diabetes care. Findings from this study provide valuable information to improve diabetes in pregnancy services to meet the needs of women during the ongoing current pandemic and beyond.

## Supplementary Information

**Additional file 1: Supplementary document 1.** Interview guide.

## Data Availability

The data that support the findings of this study are available from Monash University but restrictions apply to the availability of these data, which were used under license for the current study, and so are not publicly available. Data are however available from the authors upon reasonable request and with permission of Monash University.
