# Supplementary material for: Exploring the acceptability and experience of receiving diabetes and pregnancy care via telehealth during the COVID-19 pandemic: a qualitative study
Source: BMC Pregnancy Childbirth. 2022 Dec 13;22:932. doi: 10.1186/s12884-022-05175-z (PMC9745277; doi:10.1186/s12884-022-05175-z)
Supplement: Supplementary file 1 — Additional file 1: Supplementary document 1. Interview guide. [file 12884_2022_5175_MOESM1_ESM.docx]

***Supplementary document 1: Interview guide***

*The impact of COVID-19 and telehealth on pregnancy care*

*Ice breaker: to start off with can you please tell me about yourself, your baby and children.*

1. Can you tell me about your experiences of using telehealth while being pregnant and receiving maternity care during the COVID-19 pandemic?
   - Did your midwife/obstetrician provide appointments over the phone or on a video conference?
   - How did you feel about your face-to-face clinic visits being changed to teleconferencing and phone consultations? i.e. concerned or worried
   - For example, did you have fewer visits in person to the hospital than you expected?
   - How did you manage measuring your own blood pressure and fundal height ?
2. What made you feel reassured about your pregnancy and the health of your baby? Probe: babies’ heartbeat, ultrasounds, face-to-face visits
3. Have you used your GP for any appointments during your pregnancy, are they telehealth appointments or a phone? Has it cost you anything to attend the appoint. Why are you attending the GP?
4. During your telehealth appointments is it an issue if you don’t have the same health professional. Why?

Logistics to utilising telehealth

1. Before COVID-19 pandemic, how confident were you at using electronic devices such as mobile phone, computer, tablets/i-pads?
2. In your opinion did you received clear instructions detailing how to log on to my Telehealth appointments? How could this have been improved
3. Can you please describe anything about your experience using video conferencing/phone consultations that you found confusing or complicated? What would you prefer face to face visits, telehealth or a mixture of the 2 methods.
4. How did utilising telehealth impact upon your partner/family/ ‘tribe’ or social support during your pregnancy? Did this enable your partner to attend more appointments with you?

*Telehealth acceptability and satisfaction re: related to diabetes*

1. Can you tell me about your experience being diagnosed with diabetes during pregnancy?
   - How did you feel being diagnosed with diabetes via a phone/video consultation?
2. Can you tell me about your experience receiving diabetes education via phone or video consultations?
   - Which method of education would you have preferred when you first received your diabetes education including how to test your BSL?"
   - Following your initial GDM diagnosis, Do you prefer phone, videoconferencing or face-to-face education for such things as using a BSL monitor and lifestyle management, insulin commencement and review appointments
   - Can you talk about any disadvantages of telehealth in relation to your GDM diagnosis and management

Would you have preferred to have an initial Gestational Diabetes education session via telehealth or face to face?

- - Did you feel confident with your understanding of diabetes and how to manage your blood sugar levels?
  - Can you tell us how confident you felt testing your blood sugar levels and changing your food choices to manage your diabetes? Do you think this would have been different if you received face-to-face education?

1. If you were required to start insulin to manage your diabetes, can you tell us about your experience and feelings starting insulin via phone/video consultations?

Do you think you needed a face to face appointment if you started insulin

- - How comfortable and confident did you feel receiving this information over the phone?
  - How do you think your experience would have differed if you had had the opportunity for face-to-face clinic education?

1. Can you please tell us about the relationship you have built with your diabetes team via phone consultations?
   - Do you feel well supported by your team?
   - Did you trust your healthcare providers?
   - Do you think your relationship with your health care team would have been different if you had received face-to-face care?
   - **Can you comment on the input or diabetes education you received from the dietitian (i.e lifestyle advice)?**
   - Did you receive any written material to assist with your understanding of GDM. How did you receive this?
2. 5. If you have used interpreters, can you tell me about your experience of using interpreters during the phone/video consultations?
3. Do you think the health professionals should consider specific cultural issues during telehealth session, if so what?
4. If you have been diagnosed with diabetes during pregnancy previously, how did receiving phone consultations differ from in-person education? Which method of receiving information did you prefer and why?
5. Overall, were you satisfied and happy with the health care provided during the COVID-19 pandemic?

*Maternity health service improvements and recommendations for the future*

1. COVID has had a huge impact – what might be some of the positive aspects of this pandemic in terms of the provision of maternity care?
   - What do you think are the benefits of video conferencing and/or phone consulting over face-to-face clinic visits? (waiting times, convenience, cost)
   - If you received both phone and video consultations, did you prefer seeing your health professional on video rather than just speaking with them?
2. What could your health care provider/service have done to make your experience better?
3. Do you think maternity services should continue to deliver health care via video conferencing and/or phone consultations following the release of COVID-19 restrictions?
   - How likely would you be to recommend video conferencing or phone consultations to other pregnant women?
